# Crystal structure and Hirshfeld surface analysis of 1,6-di­amino-2-oxo-4-(thio­phen-2-yl)-1,2-di­hydro­pyridine-3,5-dicarbo­nitrile

**DOI:** 10.1107/S2056989023003237

**Published:** 2023-04-21

**Authors:** Farid N. Naghiyev, Victor N. Khrustalev, Mehmet Akkurt, Ali N. Khalilov, Ajaya Bhattarai, Fuad Sh. Kerimli, İbrahim G. Mamedov

**Affiliations:** a Department of Chemistry, Baku State University, Z. Khalilov str. 23, Az, 1148 Baku, Azerbaijan; b Peoples’ Friendship University of Russia (RUDN University), Miklukho-Maklay St. 6, Moscow, 117198, Russian Federation; c N. D. Zelinsky Institute of Organic Chemistry RAS, Leninsky Prosp. 47, Moscow, 119991, Russian Federation; d Department of Physics, Faculty of Sciences, Erciyes University, 38039 Kayseri, Türkiye; e "Composite Materials" Scientific Research Center, Azerbaijan State Economic University (UNEC), H. Aliyev str. 135, Az 1063, Baku, Azerbaijan; f Department of Chemistry, M.M.A.M.C (Tribhuvan University) Biratnagar, Nepal; University of Neuchâtel, Switzerland

**Keywords:** crystal structure, 1,2-di­hydro­pyridine, hydrogen bond, disorder, Hirshfeld surface analysis

## Abstract

The asymmetric unit of the title compound, C_11_H_7_N_5_OS, contains two independent mol­ecules (1 and 2). The thio­phene ring in mol­ecule 2 is rotationally disordered (flip disorder) by *ca* 180° (around the single C—C bond, to which it is attached) over two sites with the site-occupation factors of 0.9 and 0.1. These two orientations of the thio­phene ring in mol­ecule 2 are not equivalent. In the crystal, mol­ecules are linked by inter­molecular N—H⋯O and N—H⋯N hydrogen bonds into ribbons parallel to (022) along the *a* axis. Within the (022) planes, these ribbons are connected by van der Waals inter­actions and between the (022) planes by N—H⋯O hydrogen bonds. In mol­ecule 1, Hirshfeld surface analysis showed that the most important contributions to the crystal packing are from N⋯H/H⋯N (27.1%), H⋯H (17.6%), C⋯H/H⋯C (13.6%) and O⋯H/H⋯O (9.3%) inter­actions, while in mol­ecule 2, H⋯H (25.4%) inter­actions are the most significant contributors to the crystal packing.

## Abstract 2

In the crystal, mol­ecules are linked by inter­molecular N—H⋯O and N—H⋯N hydrogen bonds into ribbons parallel to (022) along the *a* axis. These ribbons are connected by N—H⋯O, N—H⋯N hydrogen bonds and van der Waals inter­actions.

## 1. Chemical context

The various C—C and C—N bond-formation techniques play key roles in organic synthesis (Celik *et al.*, 2023 ▸; Chalkha *et al.*, 2023 ▸; Tapera *et al.*, 2022 ▸; Lakhrissi *et al.*, 2022 ▸). The di­hydro­pyridine moiety, comprising heterocycles, demonstrates a wide spectrum of biological activities, such as anti­tumor, anti­tubercular, anti­microbial and anti-diabetic (Mohamed *et al.*, 2013 ▸; Soliman *et al.*, 2014 ▸). On the other hand, a di­hydro­pyridine scaffold is the active structural unit of a variety of natural products, drugs and functional materials. These compounds have found synthetic applications in the construction of many pharmacologically relevant natural alkaloids, such as the isoquinuclidines, ibogaine, mearsine, dioscorine, caldaphinidine D, catharanthine, vinblastine and vincristine (Sharma & Singh, 2017 ▸). 
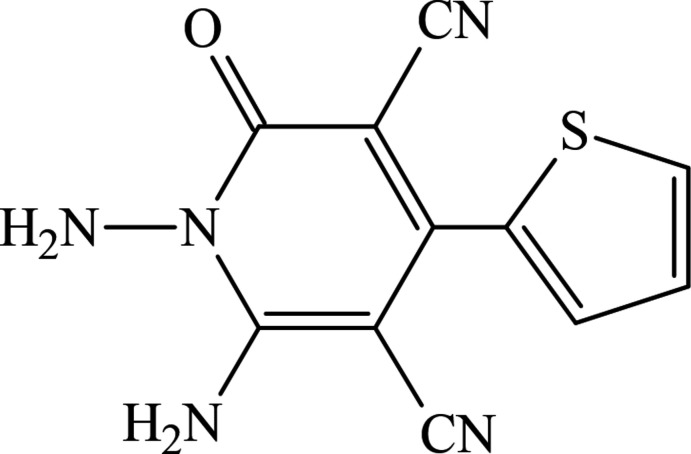


Thus, in the framework of our ongoing structural studies (Maharramov *et al.*, 2021 ▸, 2022 ▸; Naghiyev *et al.*, 2020 ▸, 2021 ▸, 2022 ▸), we report the crystal structure and Hirshfeld surface analysis of the title compound, 1,6-di­amino-2-oxo-4-(thio­phen-2-yl)-1,2-di­hydro­pyridine-3,5-dicarbo­nitrile.

## 2. Structural commentary

**Figure 1:**
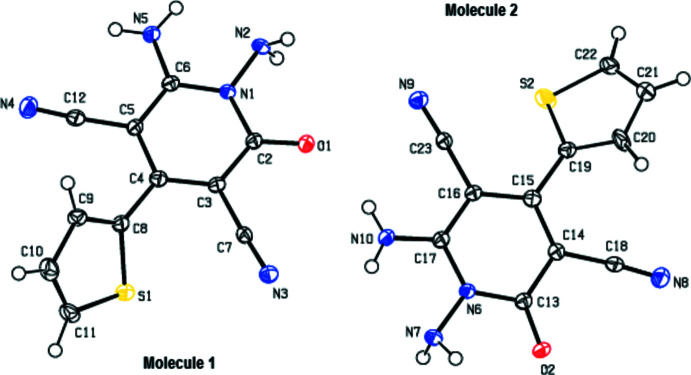
View of the two independent mol­ecules,1 and 2, in the asymmetric unit of the title compound, with displacement ellipsoids for the non-hydrogen atoms drawn at the 30% probability level. For clarity, the minor disordered components in 2 are omitted.

As seen in Fig. 1 ▸, the asymmetric unit of the title compound contains two independent mol­ecules (1 and 2). The thio­phene ring (S2′/C19/C20′–C22′) in mol­ecule 2 is rotationally disordered (flip disorder) by *ca* 180° (around the single C15—C19 bond to which it is attached) over two sites with the site-occupation factors of 0.9 and 0.1 (fixed after refinement cycles). These two orientations of the thio­phene ring in mol­ecule 2 are not equivalent.

In mol­ecule 1, the angle between the thio­phene (S1/C8–C11) and pyridine (N1/C2–C6) rings is 50.72 (9)°. In mol­ecule 2, the angle between the two disordered thio­phene rings (S2/C19–C22 and S2′/C19/C20′–C22′) is 6.2 (5)°, and they make an angle with the pyridine ring (N6/C13-C17) of 40.3 (1) and 34.8 (5)°, respectively. Mol­ecules 1 and 2 (r.m.s. deviation = 0.126 A) are almost identical and the geometric parameters are normal and comparable to those of related compounds listed in the *Database survey* section.

Mol­ecules 1 and 2 are stabilized by intra­molecular N5—H5*B*⋯N2 and N10—H10*A*⋯N7 hydrogen bonds, forming *S*(5) motifs (Table 1 ▸; Bernstein *et al.*, 1995 ▸).

## 3. Supra­molecular features and Hirshfeld surface analysis

**Figure 2:**
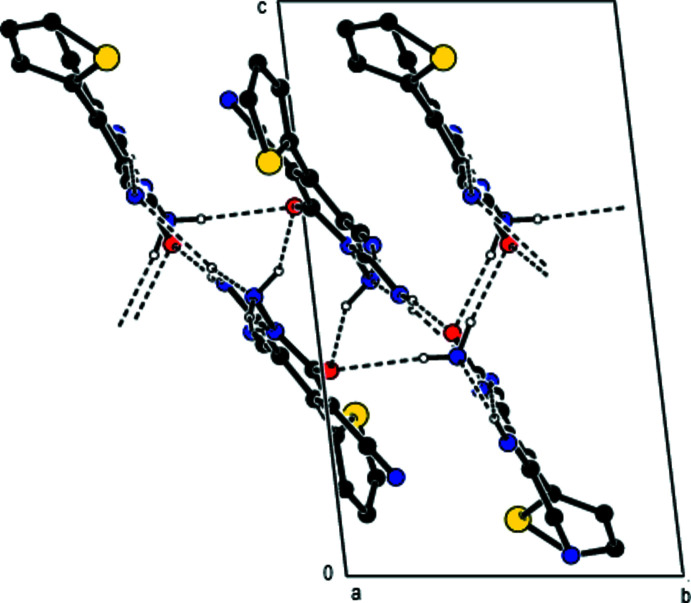
A view of the inter­molecular N—H⋯O and N—H⋯N inter­actions along the *a* axis in the crystal structure of the title compound. For clarity, H atoms not involved in hydrogen bonding and disordered components in 2 are omitted.

**Figure 3:**
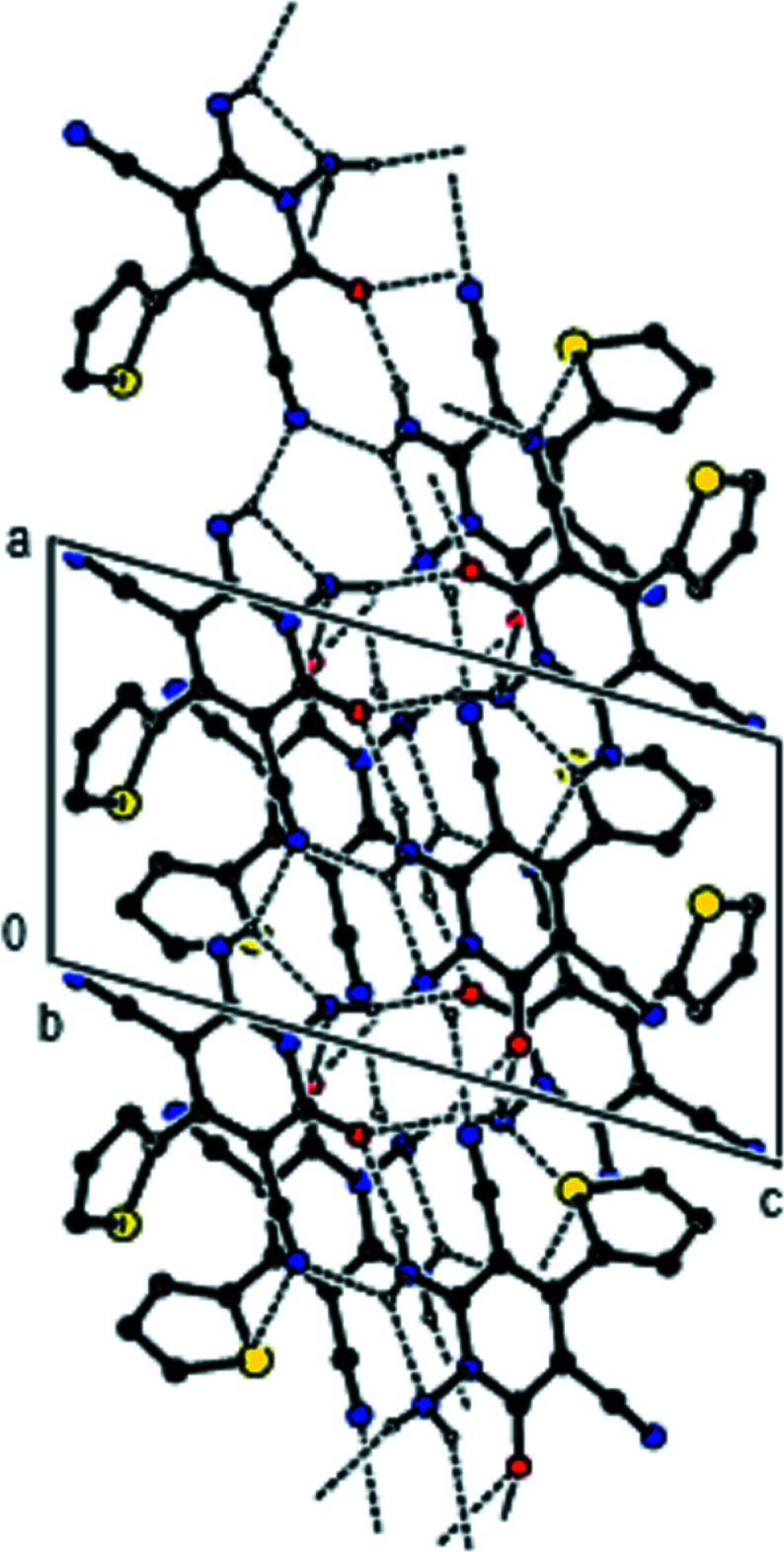
A view of the inter­molecular N—H⋯O and N—H⋯N inter­actions along the *b* axis in the crystal structure of the title compound. For clarity, H atoms not involved in hydrogen bonding and disordered components in 2 are omitted.

In the crystal, mol­ecules are linked by inter­molecular N—H⋯O and N—H⋯N hydrogen bonds into ribbons parallel to (022) along the *a*-axis (Table 1 ▸, Fig. 2 ▸). Within the (022) planes, these ribbons are connected by van der Waals inter­actions and between the (022) planes by N—H⋯O inter­molecular hydrogen bonds (Table 1 ▸, Figs. 3 ▸ and 4 ▸).

**Figure 5:**
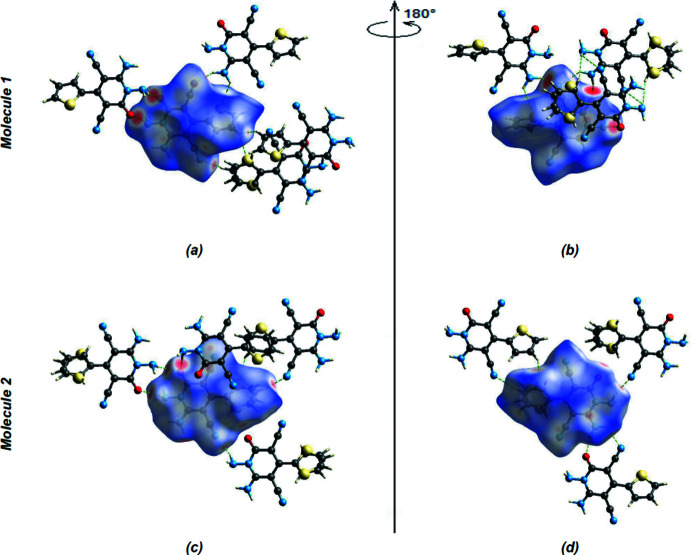
Front (*a*) and back (*b*) views of the three-dimensional Hirshfeld surface for mol­ecule 1. Front (*c*) and back (*d*) views of the three-dimensional Hirshfeld surface for mol­ecule 2. Some inter­molecular N—H⋯O and N—H⋯N inter­actions are shown.

**Figure 6:**
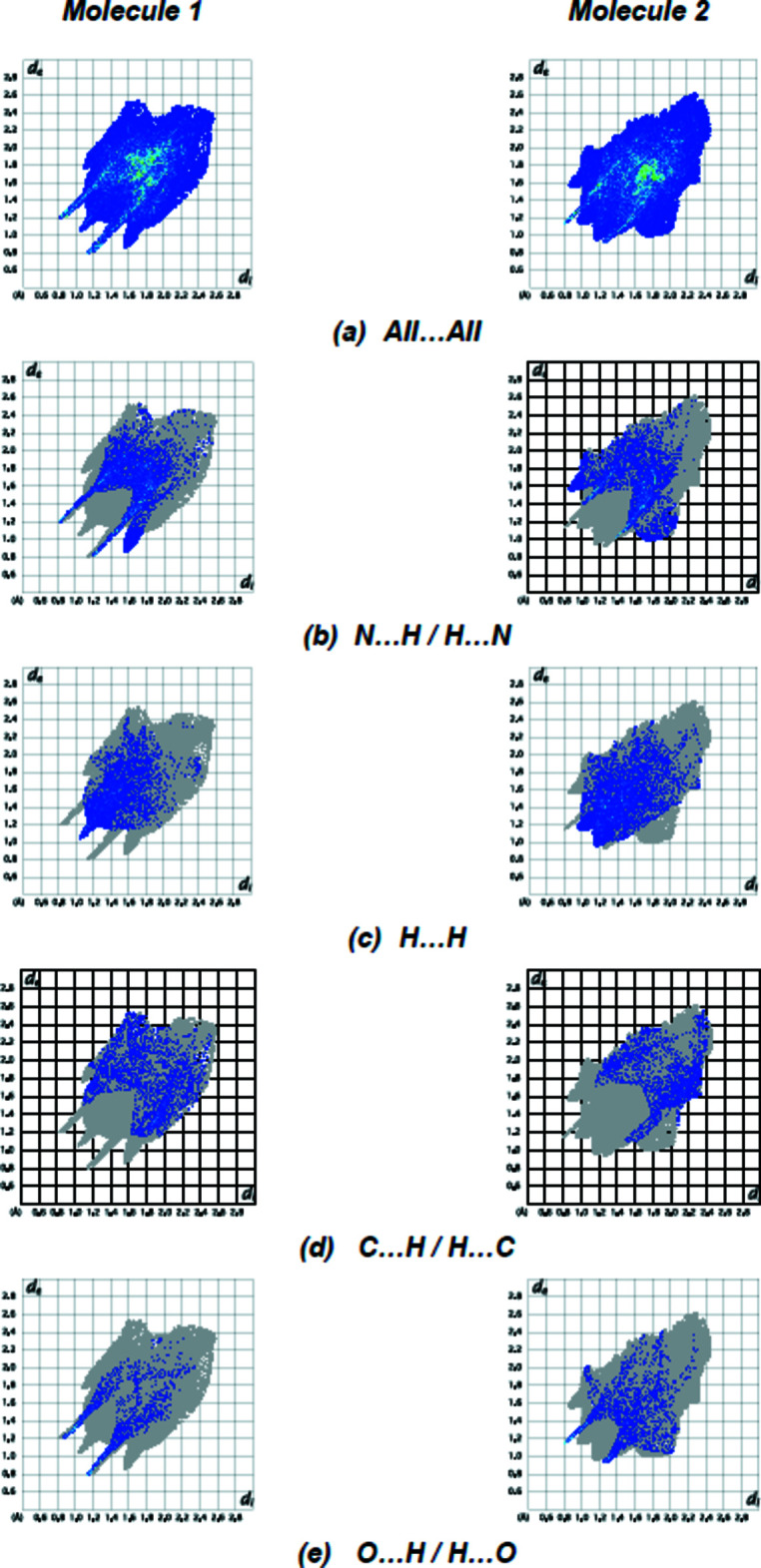
The two-dimensional fingerprint plots for mol­ecules 1 and 2 of the title compound showing (*a*) all inter­actions, and delineated into (*b*) N⋯H/H⋯N, (*c*) H⋯H, (*d*) C⋯H/H⋯C and (*e*) O⋯H/H⋯O inter­actions. The *d*_i_ and *d*_e_ values are the closest inter­nal and external distances (in Å) from given points on the Hirshfeld surface.

*CrystalExplorer17.5* (Turner *et al.*, 2017 ▸) was used to construct Hirshfeld surfaces and generate the related two-dimensional fingerprint plots to illustrate the inter­molecular inter­actions for mol­ecules 1 and 2. The *d*_norm_ mappings of 1 were conducted in the range −0.5158 to +1.0500 a.u. Bright-red circles on the *d*_norm_ surfaces (Fig. 5 ▸ *a*,*b*) represent N—H⋯O inter­action zones (Table 1 ▸). The fingerprint plots of 1 (Fig. 6 ▸) show that, while N⋯H/H⋯N (27.1%; Fig. 6 ▸ *b*) inter­actions provide the highest contribution (Table 2 ▸), as would be expected for a mol­ecule with so many H atoms, H⋯H (17.6%; Fig. 6 ▸ *c*), C⋯H/H⋯C (13.6%; Fig. 6 ▸ *d*) and O⋯H/H⋯O (9.3%; Fig. 6 ▸ *e*) contacts are also significant. Table 2 ▸ shows the contributions of all contacts. In mol­ecule 2, the *d*_norm_ mappings were performed in the range −0.5165 to +1.1535 a.u. The locations of N—H⋯O inter­actions are shown by bright-red circles on the *d*_norm_ surfaces (Fig. 5 ▸ *c*,*d*), Table 1 ▸). Fig. 6 ▸ displays the full two-dimensional fingerprint plot and those delineated into the major contacts. H⋯H inter­actions (Fig. 6 ▸ *c*; 25.4%) are the major factor in the crystal packing with N⋯H/H⋯N (Fig. 6 ▸ *b*; 24.3%), O⋯H/H⋯O (Fig. 6 ▸ *e*; 11.7%) and C⋯H/H⋯C (Fig. 6 ▸ *d*; 11.4%) inter­actions representing the next highest contributions. The percentage contributions of comparatively weaker inter­actions in mol­ecules 1 and 2 are given in Table 2 ▸. The surroundings of mol­ecules 1 and 2 are quite similar, as seen by the data comparison.

## 4. Database survey

A search of the Cambridge Structural Database (CSD, Version 5.42, update of September 2021; Groom *et al.*, 2016 ▸) gave thirteen compounds closely related to the title compounds, *viz*. CSD refcodes BEFFOL (**I**; Naghiyev *et al.*, 2022 ▸), BEFFUR (**II**; Naghiyev *et al.*, 2022 ▸), YAXQAT (**III**; Mamedov *et al.*, 2022 ▸), OZAKOS (**IV**; Naghiyev *et al.*, 2021 ▸), JEBREQ (**V**; Mohana *et al.*, 2017 ▸), JEBRAM (**VI**; Mohana *et al.*, 2017 ▸), SETWUK (**VII**; Suresh *et al.*, 2007 ▸), SETWOE (**VIII**; Suresh *et al.*, 2007 ▸), IQEFOC (**IX**; Naghiyev *et al.*, 2021 ▸a), MOKBUL (**X**; Mohamed *et al.*, 2014 ▸), PAVQIO (**XI**; Al-Said *et al.*, 2012 ▸), YIZGOE01 (**XII**; Jia & Tu, 2008 ▸) and YIBZAL (**XIII**; Eyduran *et al.*, 2007 ▸).

In the crystal of **I** (monoclinic, *C*2/*c*), pairs of mol­ecules are linked by pairs of N—H⋯N hydrogen bonds, forming dimers with an 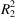 (12) ring motif. The dimers are connected by N—H⋯Br and O—H⋯O hydrogen bonds, and C—Br⋯π inter­actions, forming layers parallel to the (010) plane. Compound **II** crystallizes in the triclinic space group *P* 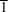 with two independent mol­ecules (**IIA** and **IIB**) in the asymmetric unit. In the crystal of **II**, mol­ecules **IIA** and **IIB** are linked by inter­molecular N—H⋯N and N—H⋯O hydrogen bonds into layers parallel to (001). These layers are connected along the *c*-axis direction by weak C—H⋯N contacts. C—H⋯π and C—N⋯π inter­actions connect adjacent mol­ecules, forming chains along the *a*-axis direction. In **III** (space group: *Pc*), the two mol­ecules in the asymmetric unit are joined together by N—H⋯O hydrogen bonds, forming a dimer with an 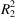 (16) ring motif. N—H⋯O and N—H⋯N hydrogen bonds link the dimers, generating chains along the *c*-axis direction, which are connected by C—Br⋯π inter­actions. In **IV** (space group: *Pc*), inter­molecular N—H⋯N and C—H⋯N hydrogen bonds, as well as N—H⋯π and C—H⋯π inter­actions, connect the mol­ecules in the crystal, generating a 3D network. In both **V** (space group: *P* 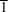 ) and **VI** (space group: *P* 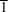 ), a supra­molecular homosynthon [ 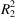 (8) ring motif] is formed through N—H⋯N hydrogen bonds. The mol­ecular structures are further stabilized by π–π stacking, and C—O⋯π, C—H⋯O and C—H⋯Cl inter­actions. In **VII** (space group: *P*2_1_/*n*), the crystal structure is stabilized by inter­molecular C—H⋯F and C—H⋯π inter­actions, and in **VIII** (space group: *P*2_1_/*c*), by inter­molecular C—H⋯O and C—H⋯π inter­actions. In **IX** (space group: *Cc*), inter­molecular N—H⋯N and C—H⋯N hydrogen bonds form mol­ecular sheets parallel to the (110) and (110) planes, crossing each other. Adjacent mol­ecules are further linked by C—H⋯π inter­actions, which form zigzag chains propagating parallel to [100]. The compound **X** (space group: *Pca*2_1_) crystallizes with two independent mol­ecules in the asymmetric unit. In the crystal, the *A* and *B* mol­ecules are linked by N—H⋯S, N—H⋯N and C—H⋯S hydrogen bonds, forming a three-dimensional network. In **XI** (space group: *P*2_1_/*c*), mol­ecules are linked into a chain along the *b*-axis direction *via* C—H⋯O inter­actions. In **XII** (space group: *P* 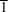 ), the crystal packing is consolidated by inter­molecular N—H⋯N, O—H⋯O and N—H⋯O hydrogen bonds. In **XIII** (space group: *P*2_1_/*c*), the mol­ecules form centrosymmetric dimers *via* N—H⋯S hydrogen bonds.

## 5. Synthesis and crystallization

The title compound was synthesized using a recently reported procedure (Babaee *et al.*, 2020 ▸), and colorless crystals were obtained upon recrystallization from an ethanol/water (3:1) solution at room temperature.

## 6. Refinement

Crystal data, data collection and structure refinement details are summarized in Table 3 ▸. The aromatic H atoms were placed at calculated positions (C—H = 0.95 Å) and refined as riding with *U*_iso_(H) = 1.2*U*_eq_(C). The N-bound H atoms were found in a difference-Fourier map, and refined freely [N2—H2*A* = 0.85 (3), N2—H2*B* = 0.93 (2), N5—H5*A* = 0.87 (3), N5—H5*B* = 0.88 (3), N7—H7*A* = 0.91 (3), N7—H7*B* = 0.88 (2), N10—H10*A* = 0.85 (2) and N10—H10*B* = 0.84 (3) Å], with *U*_iso_(H) = 1.2*U*_eq_(N). The thio­phene ring (S2/C19–C22) in mol­ecule **2** is rotationally disordered (flip disorder) by *ca* 180° (around the single C15—C19 bond, to which it is attached) over two sites with the site-occupation factors of 0.9 and 0.1 (fixed after refinement cycles). A DFIX instruction was applied to constrain the distances in the thio­phene rings of disordered mol­ecule **2**. For these rings, FLAT and EADP instructions were also used.

## Supplementary Material

Crystal structure: contains datablock(s) I. DOI: 10.1107/S2056989023003237/tx2066sup1.cif

Structure factors: contains datablock(s) I. DOI: 10.1107/S2056989023003237/tx2066Isup2.hkl

Supporting information file. DOI: 10.1107/S2056989023003237/tx2066Isup3.cml

CCDC reference: 2255359

Additional supporting information:  crystallographic information; 3D view; checkCIF report

## Figures and Tables

**Figure 4:**
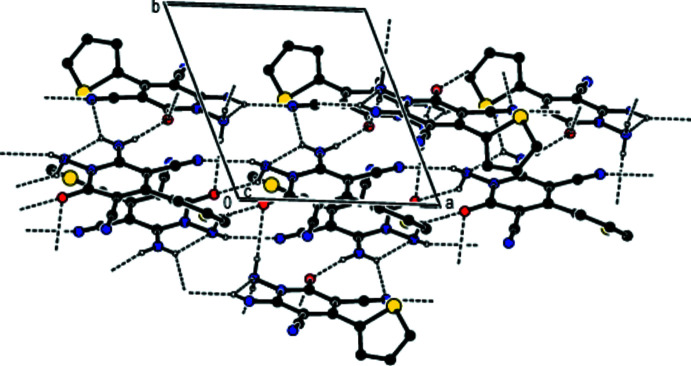
A view of the inter­molecular N—H⋯O and N—H⋯N inter­actions along the *c* axis in the crystal structure of the title compound. For clarity, H atoms not involved in hydrogen bonding and the minor disorder components in mol­ecule 2 are omitted.

**Table 1:** Hydrogen-bond geometry (Å, °)

*D*—H⋯*A*	*D*—H	H⋯*A*	*D*⋯*A*	*D*—H⋯*A*
N2—H2*A*⋯O2^i^	0.85 (3)	2.37 (3)	3.212 (2)	174 (2)
N2—H2*B*⋯O1^ii^	0.93 (2)	2.16 (3)	3.086 (2)	169 (2)
N5—H5*B*⋯N3^iii^	0.88 (3)	2.14 (3)	2.944 (2)	151 (2)
N7—H7*A*⋯O2^iv^	0.91 (3)	2.26 (2)	3.010 (2)	139 (2)
N7—H7*B*⋯N9^v^	0.88 (2)	2.55 (2)	3.348 (2)	152 (2)
N10—H10*A*⋯N3	0.85 (2)	2.62 (2)	3.196 (2)	126 (2)
N10—H10*B*⋯O1	0.84 (3)	2.11 (3)	2.921 (2)	165 (2)
N5—H5*B*⋯N2	0.88 (3)	2.22 (3)	2.606 (2)	106 (2)
N10—H10*A*⋯N7	0.85 (3)	2.23 (3)	2.626 (3)	109 (2)

Symmetry codes: (i) 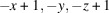 ; (ii) 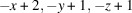 ; (iii) 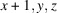 ; (iv) 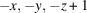 ; (v) 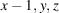 .

**Table 2:** Percentage contributions of inter­atomic contacts to the Hirshfeld surface for the title compound

Contact	Percentage contribution for mol­ecule 1	Percentage contribution for mol­ecule 2
N⋯H/H⋯N	27.1	24.3
H⋯H	17.6	25.4
C⋯H/H⋯C	13.6	11.4
O⋯H/H⋯O	9.3	11.7
C⋯C	7.3	8.5
N⋯C/C⋯N	7.0	9.0
S⋯C/C⋯S	5.4	1.3
S⋯H/H⋯S	5.1	1.8
N⋯N	2.8	2.7
S⋯N/N⋯S	2.2	1.1
O⋯C/C⋯O	1.0	1.3
S⋯S	0.8	0.3
O⋯N/N⋯O	0.7	1.2

**Table 3:** Experimental details

Crystal data	
Chemical formula	C_11_H_7_N_5_OS
*M*_r_	257.28
Crystal system, space group	Triclinic, *P* 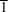
Temperature (K)	100
*a*, *b*, *c* (Å)	8.94782 (10), 9.03908 (9), 14.87299 (18)
α, β, γ (°)	90.9441 (9), 104.1197 (10), 111.7451 (10)
*V* (Å^3^)	1075.92 (2)
*Z*	4
Radiation type	Cu *K*α
μ (mm^−1^)	2.65
Crystal size (mm)	0.25 × 0.20 × 0.20

Data collection	
Diffractometer	XtaLAB Synergy, Dualflex, HyPix
Absorption correction	Multi-scan (*CrysAlis PRO*; Rigaku OD, 2021 ▸)
*T*_min_, *T*_max_	0.505, 0.561
No. of measured, independent and observed [*I* > 2σ(*I*)] reflections	32558, 4547, 4467
*R*_int_	0.033
(sin θ/λ)_max_ (Å^−1^)	0.637

Refinement	
*R*[*F* ^2^ > 2σ(*F* ^2^)], *wR*(*F* ^2^), *S*	0.040, 0.105, 1.09
No. of reflections	4547
No. of parameters	362
No. of restraints	11
H-atom treatment	H atoms treated by a mixture of independent and constrained refinement
Δρ_max_, Δρ_min_ (e Å^−3^)	0.45, −0.46

Computer programs: *CrysAlis PRO* 1.171.41.117a (Rigaku OD, 2021 ▸), *SHELXT* (Sheldrick, 2015*a* ▸), *SHELXL* (Sheldrick, 2015*b* ▸), *ORTEP-3 for Windows* (Farrugia, 2012 ▸), *PLATON* (Spek, 2020 ▸).

## supplementary crystallographic information

### Crystal data

**Table:**

C_11_H_7_N_5_OS	*Z* = 4
*M**_r_* = 257.28	*F*(000) = 528
Triclinic, *P*1	*D*_x_ = 1.588 Mg m^−^^3^
*a* = 8.94782 (10) Å	Cu *K*α radiation, λ = 1.54184 Å
*b* = 9.03908 (9) Å	Cell parameters from 26181 reflections
*c* = 14.87299 (18) Å	θ = 3.1–78.8°
α = 90.9441 (9)°	µ = 2.65 mm^−^^1^
β = 104.1197 (10)°	*T* = 100 K
γ = 111.7451 (10)°	Prism, colourless
*V* = 1075.92 (2) Å^3^	0.25 × 0.20 × 0.20 mm

### Data collection

**Table:**

XtaLAB Synergy, Dualflex, HyPix diffractometer	4467 reflections with *I* > 2σ(*I*)
Radiation source: micro-focus sealed X-ray tube	*R*_int_ = 0.033
φ and ω scans	θ_max_ = 79.3°, θ_min_ = 3.1°
Absorption correction: multi-scan (CrysAlisPro; Rigaku OD, 2021)	*h* = −11→11
*T*_min_ = 0.505, *T*_max_ = 0.561	*k* = −10→11
32558 measured reflections	*l* = −18→18
4547 independent reflections	

### Refinement

**Table:**

Refinement on *F*^2^	Secondary atom site location: difference Fourier map
Least-squares matrix: full	Hydrogen site location: mixed
*R*[*F*^2^ > 2σ(*F*^2^)] = 0.040	H atoms treated by a mixture of independent and constrained refinement
*wR*(*F*^2^) = 0.105	*w* = 1/[σ^2^(*F*_o_^2^) + (0.046*P*)^2^ + 1.11*P*] where *P* = (*F*_o_^2^ + 2*F*_c_^2^)/3
*S* = 1.09	(Δ/σ)_max_ = 0.001
4547 reflections	Δρ_max_ = 0.45 e Å^−^^3^
362 parameters	Δρ_min_ = −0.46 e Å^−^^3^
11 restraints	Extinction correction: SHELXL, Fc^*^=kFc[1+0.001xFc^2^λ^3^/sin(2θ)]^-1/4^
Primary atom site location: difference Fourier map	Extinction coefficient: 0.0028 (3)

### Special details

**Table:**

Geometry. All esds (except the esd in the dihedral angle between two l.s. planes) are estimated using the full covariance matrix. The cell esds are taken into account individually in the estimation of esds in distances, angles and torsion angles; correlations between esds in cell parameters are only used when they are defined by crystal symmetry. An approximate (isotropic) treatment of cell esds is used for estimating esds involving l.s. planes.

### Fractional atomic coordinates and isotropic or equivalent isotropic displacement parameters (Å^2^)

**Table:**

	*x*	*y*	*z*	*U*_iso_*/*U*_eq_	Occ. (<1)
S1	0.42276 (5)	0.52922 (6)	0.09865 (3)	0.02122 (13)	
O1	0.79462 (15)	0.40083 (16)	0.42272 (9)	0.0213 (3)	
N1	0.96105 (17)	0.46444 (18)	0.32428 (10)	0.0155 (3)	
N2	1.07380 (19)	0.4068 (2)	0.38163 (11)	0.0204 (3)	
H2A	1.022 (3)	0.306 (3)	0.3791 (16)	0.024*	
H2B	1.100 (3)	0.458 (3)	0.4416 (17)	0.024*	
N3	0.44940 (19)	0.4937 (2)	0.33818 (11)	0.0234 (3)	
N4	0.9777 (2)	0.6738 (2)	0.03594 (12)	0.0287 (4)	
N5	1.14206 (19)	0.5269 (2)	0.23148 (11)	0.0209 (3)	
H5A	1.166 (3)	0.560 (3)	0.1804 (18)	0.025*	
H5B	1.208 (3)	0.495 (3)	0.2738 (17)	0.025*	
C2	0.8182 (2)	0.4562 (2)	0.34957 (12)	0.0167 (3)	
C3	0.7099 (2)	0.5149 (2)	0.28660 (12)	0.0180 (3)	
C4	0.7451 (2)	0.5773 (2)	0.20585 (12)	0.0178 (3)	
C5	0.8932 (2)	0.5840 (2)	0.18606 (12)	0.0170 (3)	
C6	1.0017 (2)	0.5258 (2)	0.24648 (12)	0.0168 (3)	
C7	0.5643 (2)	0.5051 (2)	0.31343 (12)	0.0181 (3)	
C8	0.6340 (2)	0.6391 (2)	0.14265 (12)	0.0175 (3)	
C9	0.6819 (2)	0.7868 (2)	0.10847 (12)	0.0175 (3)	
H9	0.7933	0.8642	0.1235	0.021*	
C10	0.5435 (3)	0.8072 (2)	0.04835 (14)	0.0249 (4)	
H10	0.5523	0.9018	0.0193	0.030*	
C11	0.3974 (2)	0.6796 (2)	0.03603 (13)	0.0239 (4)	
H11	0.2935	0.6741	−0.0026	0.029*	
C12	0.9363 (2)	0.6369 (2)	0.10243 (13)	0.0202 (4)	
S2	0.80863 (7)	−0.08316 (7)	0.72047 (4)	0.02529 (14)	0.9
S2'	0.6895 (15)	−0.0343 (17)	0.8718 (3)	0.0314 (5)	0.1
O2	0.11544 (15)	−0.02340 (16)	0.64254 (9)	0.0214 (3)	
N6	0.31497 (17)	0.12527 (18)	0.57464 (10)	0.0160 (3)	
N7	0.19787 (19)	0.1759 (2)	0.51471 (11)	0.0193 (3)	
H7A	0.129 (3)	0.094 (3)	0.4695 (17)	0.023*	
H7B	0.140 (3)	0.197 (3)	0.5497 (16)	0.023*	
N8	0.2765 (2)	−0.1813 (2)	0.82807 (11)	0.0230 (3)	
N9	0.86800 (19)	0.19394 (19)	0.57542 (11)	0.0213 (3)	
N10	0.4975 (2)	0.25717 (19)	0.48991 (11)	0.0190 (3)	
H10A	0.424 (3)	0.292 (3)	0.4638 (16)	0.023*	
H10B	0.591 (3)	0.293 (3)	0.4796 (16)	0.023*	
C13	0.2627 (2)	0.0251 (2)	0.64097 (12)	0.0174 (3)	
C14	0.3908 (2)	−0.0122 (2)	0.70405 (12)	0.0198 (4)	
C15	0.5502 (2)	0.0312 (2)	0.69219 (12)	0.0194 (4)	
C16	0.5878 (2)	0.1196 (2)	0.61720 (12)	0.0161 (3)	
C17	0.4699 (2)	0.1710 (2)	0.55950 (12)	0.0164 (3)	
C18	0.3323 (2)	−0.1073 (2)	0.77379 (12)	0.0181 (3)	
C19	0.6737 (2)	−0.0168 (2)	0.75586 (11)	0.0189 (3)	
C20	0.6928 (6)	−0.0243 (7)	0.84874 (15)	0.0314 (5)	0.9
H20	0.6287	0.0068	0.8819	0.038*	0.9
C21	0.8164 (3)	−0.0824 (3)	0.89227 (16)	0.0287 (5)	0.9
H21	0.8463	−0.0917	0.9571	0.034*	0.9
C22	0.8866 (3)	−0.1231 (3)	0.82943 (14)	0.0211 (4)	0.9
H22	0.9689	−0.1681	0.8445	0.025*	0.9
C20'	0.7887 (14)	−0.0522 (15)	0.7247 (13)	0.02529 (14)	0.1
H20A	0.7989	−0.0491	0.6626	0.030*	0.1
C21'	0.891 (2)	−0.0945 (14)	0.8000 (13)	0.0211 (4)	0.1
H21A	0.9796	−0.1234	0.7924	0.025*	0.1
C22'	0.8558 (11)	−0.0913 (9)	0.8836 (15)	0.0287 (5)	0.1
H22A	0.9139	−0.1166	0.9398	0.034*	0.1
C23	0.7447 (2)	0.1620 (2)	0.59624 (12)	0.0170 (3)	

### Atomic displacement parameters (Å^2^)

**Table:**

	*U*^11^	*U*^22^	*U*^33^	*U*^12^	*U*^13^	*U*^23^
S1	0.0164 (2)	0.0275 (2)	0.0230 (2)	0.01097 (18)	0.00676 (16)	0.00771 (17)
O1	0.0182 (6)	0.0324 (7)	0.0191 (6)	0.0132 (5)	0.0090 (5)	0.0115 (5)
N1	0.0129 (6)	0.0205 (7)	0.0169 (7)	0.0098 (6)	0.0052 (5)	0.0070 (5)
N2	0.0155 (7)	0.0283 (9)	0.0220 (8)	0.0135 (7)	0.0048 (6)	0.0108 (6)
N3	0.0191 (7)	0.0353 (9)	0.0223 (8)	0.0156 (7)	0.0087 (6)	0.0092 (7)
N4	0.0284 (9)	0.0421 (10)	0.0286 (9)	0.0220 (8)	0.0161 (7)	0.0175 (8)
N5	0.0172 (7)	0.0323 (9)	0.0219 (8)	0.0158 (7)	0.0100 (6)	0.0119 (6)
C2	0.0156 (8)	0.0207 (8)	0.0172 (8)	0.0092 (7)	0.0069 (6)	0.0050 (6)
C3	0.0153 (8)	0.0249 (9)	0.0186 (8)	0.0113 (7)	0.0074 (6)	0.0059 (7)
C4	0.0157 (8)	0.0209 (9)	0.0197 (8)	0.0094 (7)	0.0061 (6)	0.0055 (7)
C5	0.0150 (8)	0.0211 (9)	0.0185 (8)	0.0089 (7)	0.0073 (6)	0.0077 (6)
C6	0.0139 (7)	0.0195 (8)	0.0191 (8)	0.0074 (7)	0.0064 (6)	0.0047 (6)
C7	0.0180 (8)	0.0243 (9)	0.0163 (8)	0.0123 (7)	0.0055 (6)	0.0079 (7)
C8	0.0167 (8)	0.0233 (9)	0.0183 (8)	0.0118 (7)	0.0082 (6)	0.0066 (7)
C9	0.0166 (8)	0.0180 (8)	0.0222 (8)	0.0105 (7)	0.0068 (6)	0.0037 (7)
C10	0.0322 (10)	0.0284 (10)	0.0253 (9)	0.0219 (9)	0.0107 (8)	0.0113 (8)
C11	0.0241 (9)	0.0374 (11)	0.0189 (8)	0.0221 (8)	0.0048 (7)	0.0041 (7)
C12	0.0165 (8)	0.0263 (9)	0.0241 (9)	0.0139 (7)	0.0074 (7)	0.0094 (7)
S2	0.0260 (3)	0.0395 (3)	0.0210 (3)	0.0241 (2)	0.00679 (19)	0.0055 (2)
S2'	0.0332 (9)	0.0657 (15)	0.0095 (13)	0.0340 (10)	0.0073 (14)	0.0067 (17)
O2	0.0136 (6)	0.0303 (7)	0.0236 (6)	0.0096 (5)	0.0086 (5)	0.0107 (5)
N6	0.0131 (6)	0.0201 (7)	0.0182 (7)	0.0090 (6)	0.0058 (5)	0.0076 (6)
N7	0.0159 (7)	0.0262 (8)	0.0209 (7)	0.0128 (6)	0.0058 (6)	0.0092 (6)
N8	0.0224 (8)	0.0257 (8)	0.0236 (8)	0.0100 (7)	0.0097 (6)	0.0084 (6)
N9	0.0179 (7)	0.0260 (8)	0.0243 (8)	0.0111 (6)	0.0087 (6)	0.0080 (6)
N10	0.0156 (7)	0.0251 (8)	0.0207 (7)	0.0102 (6)	0.0088 (6)	0.0101 (6)
C13	0.0157 (8)	0.0205 (9)	0.0186 (8)	0.0085 (7)	0.0066 (6)	0.0052 (6)
C14	0.0170 (8)	0.0258 (9)	0.0197 (8)	0.0104 (7)	0.0067 (7)	0.0095 (7)
C15	0.0175 (8)	0.0246 (9)	0.0196 (8)	0.0110 (7)	0.0064 (7)	0.0063 (7)
C16	0.0134 (8)	0.0194 (8)	0.0174 (8)	0.0073 (6)	0.0059 (6)	0.0040 (6)
C17	0.0159 (8)	0.0190 (8)	0.0164 (8)	0.0079 (7)	0.0064 (6)	0.0032 (6)
C18	0.0137 (7)	0.0218 (9)	0.0201 (8)	0.0087 (7)	0.0038 (6)	0.0050 (7)
C19	0.0148 (8)	0.0232 (9)	0.0220 (8)	0.0096 (7)	0.0071 (6)	0.0081 (7)
C20	0.0332 (9)	0.0657 (15)	0.0095 (13)	0.0340 (10)	0.0073 (14)	0.0067 (17)
C21	0.0218 (11)	0.0467 (13)	0.0228 (10)	0.0176 (10)	0.0075 (9)	0.0136 (9)
C22	0.0179 (9)	0.0206 (10)	0.0274 (12)	0.0108 (8)	0.0045 (9)	0.0116 (8)
C20'	0.0260 (3)	0.0395 (3)	0.0210 (3)	0.0241 (2)	0.00679 (19)	0.0055 (2)
C21'	0.0179 (9)	0.0206 (10)	0.0274 (12)	0.0108 (8)	0.0045 (9)	0.0116 (8)
C22'	0.0218 (11)	0.0467 (13)	0.0228 (10)	0.0176 (10)	0.0075 (9)	0.0136 (9)
C23	0.0171 (8)	0.0193 (8)	0.0171 (8)	0.0091 (7)	0.0055 (6)	0.0065 (6)

### Geometric parameters (Å, º)

**Table:**

S1—C11	1.713 (2)	O2—C13	1.232 (2)
S1—C8	1.7239 (18)	N6—C17	1.369 (2)
O1—C2	1.237 (2)	N6—C13	1.397 (2)
N1—C6	1.362 (2)	N6—N7	1.4192 (19)
N1—C2	1.395 (2)	N7—H7A	0.91 (3)
N1—N2	1.4148 (19)	N7—H7B	0.88 (2)
N2—H2A	0.85 (3)	N8—C18	1.147 (2)
N2—H2B	0.93 (2)	N9—C23	1.153 (2)
N3—C7	1.145 (2)	N10—C17	1.322 (2)
N4—C12	1.151 (2)	N10—H10A	0.85 (2)
N5—C6	1.325 (2)	N10—H10B	0.84 (3)
N5—H5A	0.87 (3)	C13—C14	1.442 (2)
N5—H5B	0.88 (3)	C14—C15	1.389 (2)
C2—C3	1.433 (2)	C14—C18	1.432 (2)
C3—C4	1.391 (2)	C15—C16	1.417 (2)
C3—C7	1.426 (2)	C15—C19	1.471 (2)
C4—C5	1.406 (2)	C16—C17	1.414 (2)
C4—C8	1.468 (2)	C16—C23	1.427 (2)
C5—C6	1.412 (2)	C19—C20	1.356 (2)
C5—C12	1.429 (2)	C19—C20'	1.360 (3)
C8—C9	1.388 (3)	C20—C21	1.418 (3)
C9—C10	1.415 (2)	C20—H20	0.9500
C9—H9	0.9500	C21—C22	1.360 (2)
C10—C11	1.353 (3)	C21—H21	0.9500
C10—H10	0.9500	C22—H22	0.9500
C11—H11	0.9500	C20'—C21'	1.420 (3)
S2—C22	1.702 (2)	C20'—H20A	0.9500
S2—C19	1.7120 (17)	C21'—C22'	1.359 (3)
S2'—C19	1.711 (3)	C21'—H21A	0.9500
S2'—C22'	1.719 (3)	C22'—H22A	0.9500
			
C11—S1—C8	91.74 (9)	H7A—N7—H7B	110 (2)
C6—N1—C2	124.17 (14)	C17—N10—H10A	117.1 (16)
C6—N1—N2	116.35 (13)	C17—N10—H10B	121.3 (16)
C2—N1—N2	119.48 (14)	H10A—N10—H10B	120 (2)
N1—N2—H2A	106.5 (16)	O2—C13—N6	119.40 (15)
N1—N2—H2B	105.8 (14)	O2—C13—C14	124.96 (16)
H2A—N2—H2B	111 (2)	N6—C13—C14	115.63 (15)
C6—N5—H5A	118.2 (16)	C15—C14—C18	124.60 (16)
C6—N5—H5B	119.1 (15)	C15—C14—C13	122.10 (16)
H5A—N5—H5B	123 (2)	C18—C14—C13	113.12 (15)
O1—C2—N1	118.98 (15)	C14—C15—C16	118.19 (15)
O1—C2—C3	125.41 (15)	C14—C15—C19	119.86 (15)
N1—C2—C3	115.61 (14)	C16—C15—C19	121.94 (15)
C4—C3—C7	122.82 (15)	C17—C16—C15	121.00 (15)
C4—C3—C2	122.37 (15)	C17—C16—C23	116.69 (15)
C7—C3—C2	114.82 (15)	C15—C16—C23	122.31 (15)
C3—C4—C5	118.57 (15)	N10—C17—N6	117.38 (15)
C3—C4—C8	121.73 (15)	N10—C17—C16	124.41 (16)
C5—C4—C8	119.68 (15)	N6—C17—C16	118.18 (15)
C4—C5—C6	120.31 (15)	N8—C18—C14	175.66 (18)
C4—C5—C12	123.03 (15)	C20—C19—C15	126.3 (2)
C6—C5—C12	116.49 (15)	C20'—C19—C15	120.7 (8)
N5—C6—N1	117.64 (15)	C20'—C19—S2'	113.7 (9)
N5—C6—C5	123.40 (16)	C15—C19—S2'	125.6 (3)
N1—C6—C5	118.96 (15)	C20—C19—S2	109.75 (18)
N3—C7—C3	176.99 (18)	C15—C19—S2	123.87 (12)
C9—C8—C4	126.08 (16)	C19—C20—C21	114.3 (3)
C9—C8—S1	111.33 (13)	C19—C20—H20	122.8
C4—C8—S1	122.57 (13)	C21—C20—H20	122.8
C8—C9—C10	111.33 (16)	C22—C21—C20	111.4 (2)
C8—C9—H9	124.3	C22—C21—H21	124.3
C10—C9—H9	124.3	C20—C21—H21	124.3
C11—C10—C9	113.81 (17)	C21—C22—S2	111.52 (17)
C11—C10—H10	123.1	C21—C22—H22	124.2
C9—C10—H10	123.1	S2—C22—H22	124.2
C10—C11—S1	111.78 (14)	C19—C20'—C21'	109.0 (15)
C10—C11—H11	124.1	C19—C20'—H20A	125.5
S1—C11—H11	124.1	C21'—C20'—H20A	125.5
N4—C12—C5	175.40 (18)	C22'—C21'—C20'	116 (2)
C22—S2—C19	92.90 (9)	C22'—C21'—H21A	121.9
C19—S2'—C22'	91.8 (9)	C20'—C21'—H21A	121.9
C17—N6—C13	124.25 (14)	C21'—C22'—S2'	109.3 (16)
C17—N6—N7	117.12 (14)	C21'—C22'—H22A	125.3
C13—N6—N7	118.50 (13)	S2'—C22'—H22A	125.3
N6—N7—H7A	108.6 (15)	N9—C23—C16	177.12 (19)
N6—N7—H7B	106.1 (15)		
			
C6—N1—C2—O1	−178.66 (16)	C18—C14—C15—C19	−2.0 (3)
N2—N1—C2—O1	1.2 (2)	C13—C14—C15—C19	−176.95 (17)
C6—N1—C2—C3	1.1 (3)	C14—C15—C16—C17	3.9 (3)
N2—N1—C2—C3	−179.01 (15)	C19—C15—C16—C17	−177.03 (16)
O1—C2—C3—C4	179.29 (18)	C14—C15—C16—C23	−176.47 (17)
N1—C2—C3—C4	−0.5 (3)	C19—C15—C16—C23	2.6 (3)
O1—C2—C3—C7	−0.4 (3)	C13—N6—C17—N10	175.40 (16)
N1—C2—C3—C7	179.81 (16)	N7—N6—C17—N10	−0.6 (2)
C7—C3—C4—C5	179.12 (17)	C13—N6—C17—C16	−2.9 (3)
C2—C3—C4—C5	−0.6 (3)	N7—N6—C17—C16	−178.87 (15)
C7—C3—C4—C8	0.6 (3)	C15—C16—C17—N10	178.19 (17)
C2—C3—C4—C8	−179.13 (17)	C23—C16—C17—N10	−1.5 (3)
C3—C4—C5—C6	1.1 (3)	C15—C16—C17—N6	−3.6 (3)
C8—C4—C5—C6	179.65 (16)	C23—C16—C17—N6	176.68 (15)
C3—C4—C5—C12	176.19 (17)	C14—C15—C19—C20	−36.9 (4)
C8—C4—C5—C12	−5.2 (3)	C16—C15—C19—C20	144.0 (4)
C2—N1—C6—N5	179.57 (16)	C14—C15—C19—C20'	146.3 (6)
N2—N1—C6—N5	−0.3 (2)	C16—C15—C19—C20'	−32.8 (6)
C2—N1—C6—C5	−0.7 (3)	C14—C15—C19—S2'	−33.7 (6)
N2—N1—C6—C5	179.46 (16)	C16—C15—C19—S2'	147.2 (6)
C4—C5—C6—N5	179.26 (18)	C14—C15—C19—S2	139.30 (16)
C12—C5—C6—N5	3.8 (3)	C16—C15—C19—S2	−39.8 (2)
C4—C5—C6—N1	−0.5 (3)	C22'—S2'—C19—C20	−73 (10)
C12—C5—C6—N1	−175.92 (16)	C22'—S2'—C19—C20'	0.00 (9)
C3—C4—C8—C9	129.8 (2)	C22'—S2'—C19—C15	179.99 (5)
C5—C4—C8—C9	−48.7 (3)	C22'—S2'—C19—S2	6.2 (6)
C3—C4—C8—S1	−52.1 (2)	C22—S2—C19—C20	0.9 (3)
C5—C4—C8—S1	129.31 (16)	C22—S2—C19—C20'	120 (7)
C11—S1—C8—C9	−0.62 (14)	C22—S2—C19—C15	−175.83 (17)
C11—S1—C8—C4	−178.91 (15)	C22—S2—C19—S2'	−1.9 (5)
C4—C8—C9—C10	179.27 (16)	C20'—C19—C20—C21	−6.0 (10)
S1—C8—C9—C10	1.05 (19)	C15—C19—C20—C21	177.0 (2)
C8—C9—C10—C11	−1.1 (2)	S2'—C19—C20—C21	102 (10)
C9—C10—C11—S1	0.6 (2)	S2—C19—C20—C21	0.3 (5)
C8—S1—C11—C10	0.01 (15)	C19—C20—C21—C22	−1.8 (5)
C17—N6—C13—O2	−172.12 (17)	C20—C21—C22—S2	2.4 (3)
N7—N6—C13—O2	3.8 (2)	C19—S2—C22—C21	−1.95 (19)
C17—N6—C13—C14	8.5 (2)	C20—C19—C20'—C21'	2.8 (8)
N7—N6—C13—C14	−175.61 (15)	C15—C19—C20'—C21'	−179.99 (8)
O2—C13—C14—C15	172.61 (18)	S2'—C19—C20'—C21'	0.01 (13)
N6—C13—C14—C15	−8.0 (3)	S2—C19—C20'—C21'	−60 (7)
O2—C13—C14—C18	−2.9 (3)	C19—C20'—C21'—C22'	−0.01 (19)
N6—C13—C14—C18	176.52 (15)	C20'—C21'—C22'—S2'	0.01 (18)
C18—C14—C15—C16	177.10 (17)	C19—S2'—C22'—C21'	−0.01 (11)
C13—C14—C15—C16	2.2 (3)		

### Hydrogen-bond geometry (Å, º)

**Table:**

*D*—H···*A*	*D*—H	H···*A*	*D*···*A*	*D*—H···*A*
N2—H2*A*···O2^i^	0.85 (3)	2.37 (3)	3.212 (2)	174 (2)
N2—H2*B*···O1^ii^	0.93 (2)	2.16 (3)	3.086 (2)	169 (2)
N5—H5*B*···N3^iii^	0.88 (3)	2.14 (3)	2.944 (2)	151 (2)
N7—H7*A*···O2^iv^	0.91 (3)	2.26 (2)	3.010 (2)	139 (2)
N7—H7*B*···N9^v^	0.88 (2)	2.55 (2)	3.348 (2)	152 (2)
N10—H10*A*···N3	0.85 (2)	2.62 (2)	3.196 (2)	126 (2)
N10—H10*B*···O1	0.84 (3)	2.11 (3)	2.921 (2)	165 (2)
N5—H5*B*···N2	0.88 (3)	2.22 (3)	2.606 (2)	106 (2)
N10—H10*A*···N7	0.85 (3)	2.23 (3)	2.626 (3)	109 (2)

Symmetry codes: (i) −*x*+1, −*y*, −*z*+1; (ii) −*x*+2, −*y*+1, −*z*+1; (iii) *x*+1, *y*, *z*; (iv) −*x*, −*y*, −*z*+1; (v) *x*−1, *y*, *z*.

## References

[bb1] Al-Said, M. S., Ghorab, M. M., Ghabbour, H. A., Arshad, S. & Fun, H.-K. (2012). *Acta Cryst.* E**68**, o1679. 10.1107/S1600536812019927 PMC3379275 22719473

[bb2] Babaee, S., Zarei, M., Sepehrmansourie, H., Zolfigol, M. A. & Rostamnia, S. (2020). *ACS Omega*, **5**, 6240–6249. 10.1021/acsomega.9b02133 PMC7114146 32258858

[bb3] Bernstein, J., Davis, R. E., Shimoni, L. & Chang, N.-L. (1995). *Angew. Chem. Int. Ed. Engl.* **34**, 1555–1573.

[bb4] Çelik, M. S., Çetinus, A., Yenidünya, A. F., Çetinkaya, S. & Tüzün, B. (2023). *J. Mol. Struct.* **1272**, 134158.

[bb5] Chalkha, M., Ameziane el Hassani, A., Nakkabi, A., Tüzün, B., Bakhouch, M., Benjelloun, A. T., Sfaira, M., Saadi, M., Ammari, L. E. & Yazidi, M. E. (2023). *J. Mol. Struct.* **1273**, 134255.

[bb6] Eyduran, F., Özyürek, C., Dilek, N., Ocak Iskeleli, N. & Şendil, K. (2007). *Acta Cryst.* E**63**, o2415–o2417.

[bb7] Farrugia, L. J. (2012). *J. Appl. Cryst.* **45**, 849–854.

[bb8] Groom, C. R., Bruno, I. J., Lightfoot, M. P. & Ward, S. C. (2016). *Acta Cryst.* B**72**, 171–179. 10.1107/S2052520616003954 PMC4822653 27048719

[bb9] Jia, R. & Tu, S. J. (2008). *Acta Cryst.* E**64**, o1578. 10.1107/S1600536808022551 PMC2962196 21203277

[bb10] Lakhrissi, Y., Rbaa, M., Tuzun, B., Hichar, A., Anouar, H., Ounine, K., Almalki, F., Hadda, T. B., Zarrouk, A. & Lakhrissi, B. (2022). *J. Mol. Struct.* **1259**, 132683.

[bb11] Maharramov, A. M., Shikhaliyev, N. G., Zeynalli, N. R., Niyazova, A. A., Garazade, Kh. A. & Shikhaliyeva, I. M. (2021). *UNEC J. Eng. Appl. Sci.* **1**, 5–11.

[bb12] Maharramov, A. M., Suleymanova, G. T., Qajar, A. M., Niyazova, A. A., Ahmadova, N. E., Shikhaliyeva, I. M., Garazade, Kh. A., Nenajdenko, V. G. & Shikaliyev, N. G. (2022). *UNEC J. Eng. Appl. Sci.* **2**, 64–73.

[bb13] Mamedov, I. G., Khrustalev, V. N., Akkurt, M., Novikov, A. P., Asgarova, A. R., Aliyeva, K. N. & Akobirshoeva, A. A. (2022). *Acta Cryst.* E**78**, 291–296. 10.1107/S2056989022001232 PMC8900508 35371550

[bb14] Mohamed, S. K., Akkurt, M., Singh, K., Hussein, B. R. M. & Albayati, M. R. (2014). *Acta Cryst.* E**70**, o993–o994. 10.1107/S1600536814018029 PMC4186145 25309298

[bb15] Mohamed, S. K., Soliman, A. M., El Remaily, M. A. A. & Abdel-Ghany, H. (2013). *J. Heterocycl. Chem.* **50**, 1425–1430.

[bb16] Mohana, M., Thomas Muthiah, P. & Butcher, R. J. (2017). *Acta Cryst.* C**73**, 536–540. 10.1107/S2053229617008798 28677605

[bb17] Naghiyev, F. N., Akkurt, M., Askerov, R. K., Mamedov, I. G., Rzayev, R. M., Chyrka, T. & Maharramov, A. M. (2020). *Acta Cryst.* E**76**, 720–723. 10.1107/S2056989020005381 PMC7199244 32431939

[bb18] Naghiyev, F. N., Khrustalev, V. N., Novikov, A. P., Akkurt, M., Rzayev, R. M., Akobirshoeva, A. A. & Mamedov, I. G. (2022). *Acta Cryst.* E**78**, 554–558. 10.1107/S2056989022004741 PMC9431780 36072149

[bb19] Naghiyev, F. N., Pavlova, A. V., Khrustalev, V. N., Akkurt, M., Khalilov, A. N., Akobirshoeva, A. A. & Mamedov, İ. G. (2021). *Acta Cryst.* E**77**, 930–934. 10.1107/S2056989021007994 PMC8423002 34584764

[bb20] Rigaku OD (2021). *CrysAlis PRO*. Rigaku Oxford Diffraction, Yarnton, England.

[bb21] Sharma, V. K. & Singh, S. K. (2017). *RSC Adv.* **7**, 2682–2732.

[bb22] Sheldrick, G. M. (2015*a*). *Acta Cryst.* A**71**, 3–8.

[bb23] Sheldrick, G. M. (2015*b*). *Acta Cryst.* C**71**, 3–8.

[bb24] Soliman, A. M., Mohamed, S. K., El-Remaily, M. A. A. & Abdel-Ghany, H. (2014). *J. Heterocycl. Chem.* **51**, 1202–1209.

[bb25] Spek, A. L. (2020). *Acta Cryst.* E**76**, 1–11. 10.1107/S2056989019016244 PMC6944088 31921444

[bb26] Suresh, J., Suresh Kumar, R., Perumal, S., Mostad, A. & Natarajan, S. (2007). *Acta Cryst.* C**63**, o141–o144. 10.1107/S0108270107000224 17284810

[bb27] Tapera, M., Kekeçmuhammed, H., Tüzün, B., Sarıpınar, E., Koçyiğit, M., Yıldırım, E., Doğan, M. & Zorlu, Y. (2022). *J. Mol. Struct.* **1269**, 133816.

[bb28] Turner, M. J., McKinnon, J. J., Wolff, S. K., Grimwood, D. J., Spackman, P. R., Jayatilaka, D. & Spackman, M. A. (2017). CrystalExplorer17. The University of Western Australia.

